# A Lifestyle Monitoring System for Older Adults Living Independently Using Low-Resolution Smart Meter Data

**DOI:** 10.3390/s24113662

**Published:** 2024-06-05

**Authors:** Bhekumuzi M. Mathunjwa, Yu-Fen Chen, Tzung-Cheng Tsai, Yeh-Liang Hsu

**Affiliations:** 1 Gerontechnology Research Center, Yuan Ze University, Taoyuan 320315, Taiwan; 2 Taiwan Power Research Institute, Taipei 100046, Taiwan; 3 Industrial Technology Research Institute, Hsinchu 310401, Taiwan

**Keywords:** older adults, lifestyle monitoring, smart meter data, independent living, activity, regularity

## Abstract

Background: Monitoring the lifestyles of older adults helps promote independent living and ensure their well-being. The common technologies for home monitoring include wearables, ambient sensors, and smart household meters. While wearables can be intrusive, ambient sensors require extra installation, and smart meters are becoming integral to smart city infrastructure. Research Gap: The previous studies primarily utilized high-resolution smart meter data by applying Non-Intrusive Appliance Load Monitoring (NIALM) techniques, leading to significant privacy concerns. Meanwhile, some Japanese power companies have successfully employed low-resolution data to monitor lifestyle patterns discreetly. Scope and Methodology: This study develops a lifestyle monitoring system for older adults using low-resolution smart meter data, mapping electricity consumption to appliance usage. The power consumption data are collected at 15-min intervals, and the background power threshold distinguishes between the active and inactive periods (0/1). The system quantifies activity through an active score and assesses daily routines by comparing these scores against the long-term norms. Key Outcomes/Contributions: The findings reveal that low-resolution data can effectively monitor lifestyle patterns without compromising privacy. The active scores and regularity assessments calculated using correlation coefficients offer a comprehensive view of residents’ daily activities and any deviations from the established patterns. This study contributes to the literature by validating the efficacy of low-resolution data in lifestyle monitoring systems and underscores the potential of smart meters in enhancing elderly people’s care.

## 1. Introduction

Smart meter applications have evolved significantly, offering multifaceted benefits beyond conventional energy monitoring. Initially designed to facilitate more efficient energy use, smart meters now play a pivotal role in integrating with Internet of Things (IoTs) devices and systems in many research projects [1,2,3], thereby enabling innovative home automation solutions. Furthermore, smart meters serve as foundational components of the Internet of Things (IoTs) ecosystem, seamlessly integrating with other IoT devices and systems to enable innovative home automation solutions. By leveraging the data collected from smart meters, IoT devices can optimize energy usage, automate tasks, and enhance the overall comfort and convenience for homeowners [1,4,5]. This integration opens up possibilities for intelligent energy management systems that dynamically respond to changing energy demands and environmental conditions, paving the way for a more sustainable and connected future [6,7]. With real-time data collection and analysis capabilities, smart meters empower users to make informed decisions about their energy consumption patterns, leading to improved energy efficiency and cost savings [8,9,10].

Non-Intrusive Appliance Load Monitoring (NIALM), initially proposed in [11], utilizes supervised learning algorithms to disaggregate total household electricity consumption into individual appliance usage patterns without requiring additional sensors or intrusive monitoring devices [12,13,14]. While NIALM offers valuable insights into household energy consumption patterns by analyzing the distinctive electrical signatures of various appliances, it raises significant privacy concerns as it involves the detailed monitoring of individuals’ activities within their homes [15,16,17,18]. The high-resolution nature of NIALM can reveal sensitive information about users, prompting the need for robust privacy safeguards and data protection measures.

In Japan, smart meter-based monitoring systems for older adults have become integral components of home energy management strategies, prioritizing energy conservation, electrical safety, and the well-being of elderly individuals living independently. Japan has one of the fastest aging populations globally, making it an ideal context for studying innovative solutions to support the well-being of older adults living independently. The country faces unique challenges related to an aging society, such as increasing healthcare costs and a shrinking workforce, driving the need for effective monitoring systems to ensure the safety and health of older individuals. With the deregulation of the electricity industry in 2016, Japanese power companies have introduced tailored home care services to address market competition and societal aging. These systems, deployed by various electric power companies, utilize low-resolution smart meter data collected at a frequency of 30 min to analyze the changes in lifestyle patterns directly from electricity usage. Initiatives, such as Tokyo Electric Power Company’s (TEPCO), Tokyo, Japan “Anshin Plan for Long-Distance Families”, Kyushu Electric Power Company’s (Kyushu, Japan) “Kyuden Anshin Support” plan, Chubu Electric Power Company’s (Chubu, Japan) “Monitoring Assistance Service”, and Kansai Electric Power Company’s (Kansai, Japan) “Happy Protector” service, utilize smart meter data to monitor the changes in electricity consumption patterns, detect abnormal usage, establish daily life rhythms, and issue notifications for deviations in routine. Through unsupervised learning techniques, they establish indicators, such as electricity thresholds, routine models, and upper and lower limits, based on electricity usage over the last 7, 14, and 30 days. Caregivers receive notifications when daily electricity usage indicators deviate from the norm, enhancing their ability to respond to older people’s routine changes, which might be associated with a possible decline in health [17]. However, this approach relies entirely on power consumption data and does not map to appliance usage, raising privacy concerns. The increasing integration of smart meter technology in home energy management systems represents a significant advancement in how we can support older adults living independently. As Japan faces a rapidly aging population, there is a pressing need to develop non-intrusive, scalable solutions that ensure the safety and well-being of elderly people, while maximizing their ability to live independently. Smart meters, which primarily collect low-resolution power consumption data, offer a unique opportunity to monitor daily routines and detect any abnormal activities without using more invasive methods. This study aims to explore the effectiveness of these systems in real-world settings, evaluating their potential to assist caregivers by providing timely alerts about significant changes in electricity usage patterns. This data-driven approach not only promises to enhance quality of care, but also ensures timely interventions, ultimately supporting the health and safety of older adults. By focusing on the capabilities of smart meter technology, we address critical challenges, such as increasing healthcare costs and the shrinking workforce, associated with societal aging. Detecting the deviations in electricity usage patterns among older adults living independently is highly important for caregivers and family members. Changes in energy consumption can signal shifts in routine, lifestyle, or health status. For instance, sudden increases or decreases might suggest alterations in daily activities, mobility issues, or the onset of health problems [19]. By monitoring these patterns, caregivers can intervene and address long-term health concerns. Therefore, leveraging smart meter technology to track electricity usage offers a proactive and non-intrusive approach to safeguarding the well-being of older adults, supporting the need for efficient remote monitoring and caregiving in aging populations [20].

This study leverages smart meter data from the Taiwan Power Company (TPC), Taipei, Taiwan. The TPC Smart Meter Initiative entails the deployment of advanced metering infrastructure across 1.2 million households, offering a comprehensive platform for detailed energy consumption analysis. With a data frequency of 15 min, the smart meters collect and transmit electricity usage data, facilitating the monitoring and analysis of energy consumption patterns. This large-scale initiative represents a concerted effort to enhance lifestyle monitoring and energy management, leveraging the capabilities of smart meter technology to promote efficiency and sustainability in households across Taiwan.

This study applies Taiwan Power Company’s (TPC) smart electricity meter data to the daily routine analysis of older adults. Like the service models of various power companies in Japan, this research aims to develop a lifestyle monitoring system using low-resolution smart meter data for older adults living independently at home. Power consumption data are acquired every 15 min using the TPC smart meter. The data are mapped to appliance usage (0/1), which often relates to older adult’s instrumental activities of daily living (IADL). The indices of “activity” and “regularity” of appliance usage based on comparison with the pattern of the last 28 days are calculated. A prototype dashboard is developed to display the users’ indices of activity and regularity.

This paper is structured as follows: Section 1 provides an introduction to the research. Section 2 details the methodology, including smart meter data extraction and routine pattern evaluation. Section 3 presents the results. Section 4 presents the discussion, and Section 5 provides the conclusions and future work.

## 2. Materials and Methods

Monitoring the lifestyle of older adults using changes in power consumption data involves a systematic process aimed at identifying patterns, anomalies, and trends in energy usage for a day [21,22,23]. This process entails several essential steps: smart meter data extraction [24], activity detection by distinguishing between electricity consumption due to constant appliance usage (background power) and user-specific appliance usage [25], quantifying the user’s daily activity by calculating active scores, comparing the user’s data for the day with norm data for the last 28 days, and assessing the regularity of the user’s daily lifestyle.

### 2.1. Data Collection and Storage

Smart meter data extraction involves retrieving power consumption data from users’ homes through smart meters installed on their premises. Power consumption data for this study are collected from 125 households using the TPC smart meter, which is equipped to record power consumption at 15-min intervals. The electricity consumption data are stored in the TPC database, facilitating easy access and retrieval. Electric consumption data are processed and converted upon retrieval into a low-resolution format (0/1) to ensure user privacy and security. Access to the data is facilitated through the Application Programming Interface (API). The motivation behind selecting this particular sample lies in its alignment with the study’s objectives and target population. The users included in the sample are over 65 years old, reflecting the demographic of interest in understanding the lifestyle patterns of older adults. Furthermore, the users primarily live alone, with the presence of children caregivers who read the data, and the inclusion of some users who have caregivers who visit them from time to time. However, the limitations include the fact that the sample may not fully represent the entire older adult population, as it may exclude individuals who live in assisted living facilities or have different caregiving arrangements, which could limit the generalizability of the study’s findings to older adults in other living situations.

### 2.2. Establishing a Background Power Threshold

Household appliances exhibit distinct power characteristics, as delineated in Table 1. Appliances such as microwave ovens manifest a high wattage and a short usage duration, resulting in sharp peaks in power consumption. Conversely, appliances like televisions display a lower wattage and longer usage times, leading to less pronounced power consumption curves. Due to varying appliance behaviors, the direct interpretation of power consumption curves can be challenging, especially using low-resolution power consumption data. This study converts power consumption data into an appliance usage format (0/1) to facilitate the analysis of daily routines by establishing a background power threshold.

Background power, also known as the base load, is influenced by continuous power sources and may vary with seasonal changes. Determining background power is crucial for understanding power consumption patterns, especially when monitoring the lifestyle of older adults. A study by Kim and Park [25] monitored power consumption for a day when the user was not at home and used it to establish background power. The determination of the threshold for background power is a pivotal step in discerning between user-specific electricity consumption and constant appliance usage (e.g., refrigerator).

**Figure 1:**
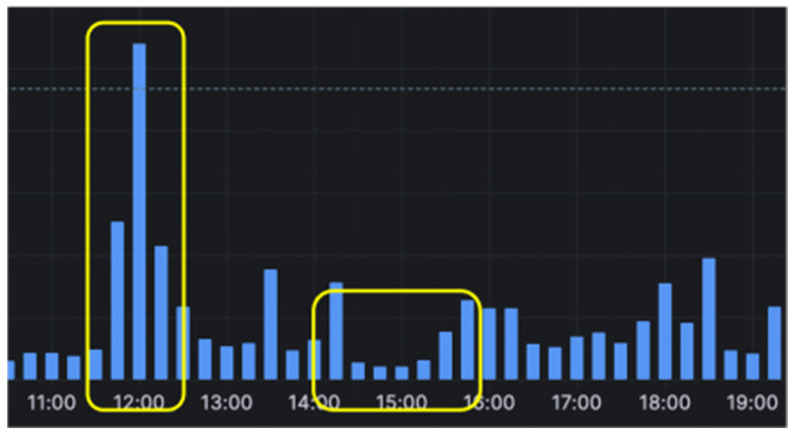
Bar chart of electricity usage every 15 min for a certain household. The yellow boxes shows the high peaks (appliance usage) and low valleys (no appliance usage), respectively.

This study employs an unsupervised learning method to establish a threshold for the user’s background power, chosen for its suitability in scenarios where labeled data may be scarce or difficult to obtain. The approach takes the mean for low valleys in the power consumption data as the threshold for background power. Electricity consumption for the day is compared with the threshold and converted into appliance usage (0/1) to monitor electricity appliance usage. This approach does not require labeled examples, making it ideal for analyzing complex datasets like household electricity consumption data. Unsupervised learning methods automatically identify patterns and structures in the data without explicit supervision, which is advantageous given the variability in household electricity consumption patterns [26,27]. Additionally, these methods offer flexibility in handling different data distributions and can adapt to changes over time, aligning with this study’s objective of distinguishing between user-specific electricity consumption and constant appliance usage. Figure 1 shows a bar chart of power consumption every 15 min for a household, where high peaks often represent appliance usage, while flat, low valleys are more likely to indicate no appliance usage (background power).

#### 2.2.1. Threshold Determination

This algorithm identifies low valleys in electricity consumption data and calculates their mean, thereby establishing a baseline for distinguishing appliance usage from background power (Algorithm 1).
**Algorithm 1**: Threshold for Background PowerInput: Power consumption data represented by a vector “A” containing energy usage values in 15-min intervals.Initialization:Create an empty array “dif” to store the differences between adjacent elements.Initialize variables “sum” and “count” to calculate the sum and count of elements in low valleys.3.Calculate Differences:Iterate over the elements of A from index 1 to len(A) − 2:
a.If i = 0, calculate dif[i] = max(A[i] − A[i + 1], 0).b.If i = len(A) − 1, calculate dif[i] = max(A[i] − A[i − 1], 0).c.Otherwise, calculate dif[i] = max(A[i] − A[i − 1], 0) + max(A[i] − A[i + 1], 0).4.Calculate Mean for Low Valleys:Iterate over the elements of A from index 1 to len(A) − 2:
a.If dif[i] = 0, add A[i] to sum and increment count.
5.Calculate Mean:Calculate the mean value of the elements identified as low valleys.6.Output: Return the mean value as the threshold for background power.

**Figure 2:**
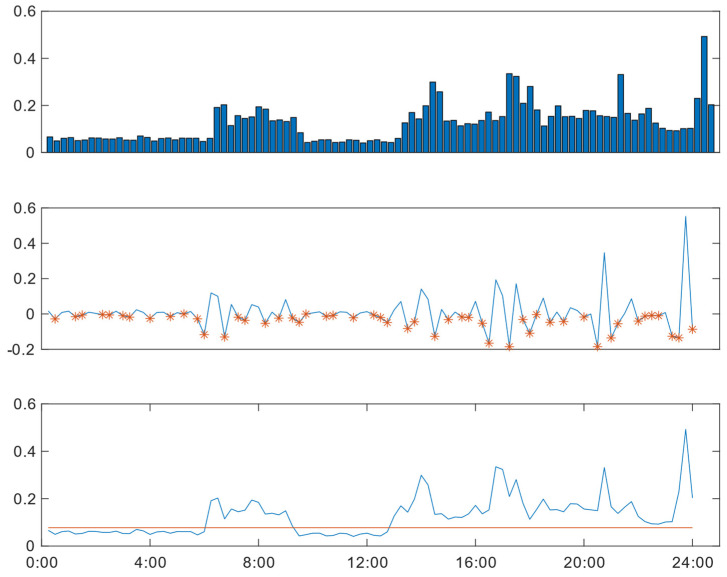
Threshold determination including power consumption data for the day (upper figure), the difference between power values (asterisks in the middle figure), and the threshold for the day (horizontal line in the lower figure).

An example illustrating the application of the algorithm in finding the threshold is depicted in Figure 2. The top figure displays the power consumption data for the day. The middle part of the figure represents the differences between the values used to identify the low valley positions in the power consumption data, indicated by the asterisks. The power consumption data in the low valley positions are averaged to derive the threshold for the day (horizontal line), as illustrated in the lower part of the figure.

#### 2.2.2. Appliance Usage Identification

**Figure 3:**
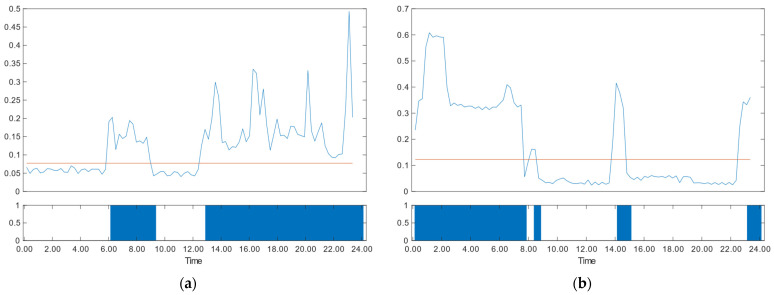
Power consumption data (blue lines) converted to household appliance usage using the threshold (red lines) for two homes. (**a**) Home A with active score: 57.3; (**b**) Home B with active score: 43.8.

Once the household’s appliance usage threshold is determined, a comparison between the electricity consumption data for the day and the threshold is made, categorizing the intervals as active (1) or inactive (0). Figure 3a,b illustrate this process for two homes (Home A and Home B), with the vertical axis representing power consumption and the horizontal axis denoting time in 15-min intervals. The upper part of the figures displays the threshold usage (red line) on the power consumption data. For power consumption higher than the threshold, “1” is returned, indicating “active”, and “0” is returned for power consumption lower than the threshold, indicating “inactive”, as shown in the lower part of the figure. This comprehensive approach effectively distinguishes between appliance usage and background power, enabling the insightful analysis of household energy consumption patterns.

### 2.3. Quantifying Daily Activity and Self-Comparison

The active score and norm establishment process serve as fundamental tools for quantifying household appliance usage levels and establishing standard models for routine assessment. Power consumption data for the day are mapped onto 96 data points (15 min each), indicating appliance usage (0/1) and the older adult’s daily living activity. The active score is calculated by finding the percentage of “active” (1s) in the 96 data points of the day as per Equation (1).

(1) $$AS=100\times \frac{1}{96}\sum_{i=1}^{96}A_{i}$$

where $A_{i}$ represents appliance usage.

Self-comparison is made between the appliance usage data for the day and the appliance usage norm data of the last 28 days to understand whether their lifestyle deviates from the long-term norm. The appliance usage norm is calculated daily by finding the percentage of “active” (1s) in each period of the day (15-min intervals) in the 28 days. The appliance usage norm is calculated for the 96 data points daily. The norm score is calculated by finding the mean active scores for the previous 28 days’ data, as shown in Equation (2).

(2) $${Norm}_{4w}=\frac{\sum_{i=1}^{28}{AS}_{i}}{28}$$

where ${AS}_{i}$ represents the active score for each day in the 28 days. Low and high norms are also calculated daily from the 28-day data by sorting the active scores in ascending order, and then obtaining each half’s average (14 days).

### 2.4. Regularity Assessment

The regularity of the users’ lifestyle is also assessed by calculating the correlation coefficients (CCs) of the previous day’s appliance usage data with the appliance usage norm of the 28 days. This statistical measure allows us to quantify the degree of similarity between the daily appliance usage and the appliance usage norms for the last 28 days. The CC is calculated using Equation (3).

(3) $$CC=\frac{n\sum XY-\sum X\sum Y}{\sqrt{\left(n\sum X^{2}-{\left(\sum X\right)}^{2})(n\sum Y^{2}-{\left(\sum Y\right)}^{2}\right)}}$$

where $X_{i}$ and $Y_{i}$ represent the appliance usage for the day and the appliance usage norm, respectively. The correlation coefficient ranges from −1 to 1, where “1” indicates a perfect positive correlation, meaning the daily routines perfectly match the norm. The value “−1“ indicates a perfect negative correlation, meaning the daily routine is the exact opposite of the norms. The value “0” indicates no correlation, implying no similarity between the daily routines and the norms.

## 3. Results

### 3.1. Background Power Threshold Establishment and Accuracy Verification

**Figure 4:**
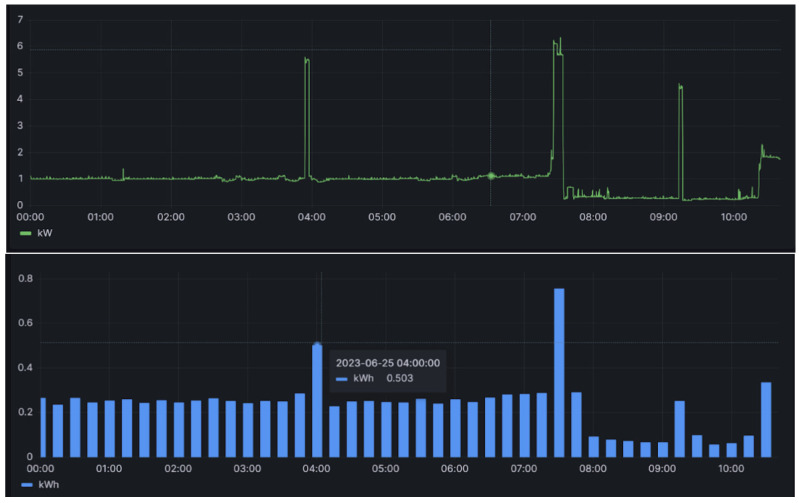
Household’s third-party electricity meter data every 10 s and corresponding electricity consumption data every 15 min.

**Figure 5:**
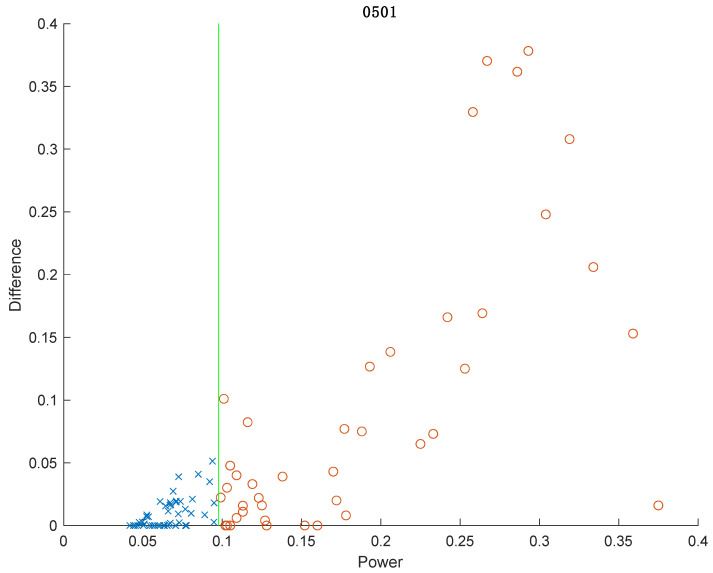
Determining appliance usage in 96 periods of a household in a day, with a background power threshold accuracy of 98.9%. The green line represents the background power threshold, “x” indicates no appliance usage, and “o” indicates appliance usage.

This section details establishing and verifying the background power threshold, building upon the methodology outlined in the preceding section. This step is pivotal in utilizing electricity consumption data for the analysis of activity and regularity, achieved by calculating the background power threshold and categorizing electricity consumption into usage (0/1) based on the threshold. Figure 4 shows an example of power consumption data from a third-party meter collected at 10-s intervals (upper part) and the corresponding power consumption aggregated to 15-min intervals (lower part). The manual determination of appliance usage within 15 min is facilitated by aligning these data with records obtained every 10 s, where “x” denotes no appliance usage and “o” indicates usage, as illustrated in Figure 5. Notably, the intervals lacking appliance usage predominantly cluster in the lower left part of the graph, which is indicative of low electricity consumption and a small difference. Another observation is that many periods fall on the horizontal axis, indicating a “difference” of 0, which represents the aforementioned “electricity valley”. However, this also includes some periods of appliance usage, marked as “o”. This study computes the average electricity consumption of all the valley points over the day, defining this as the background power threshold (green line in Figure 5), which is updated daily. The accuracy of appliance usage determination with this threshold reaches 98.9%, with only one misjudgment observed, as illustrated in Figure 5.

**Figure 6:**
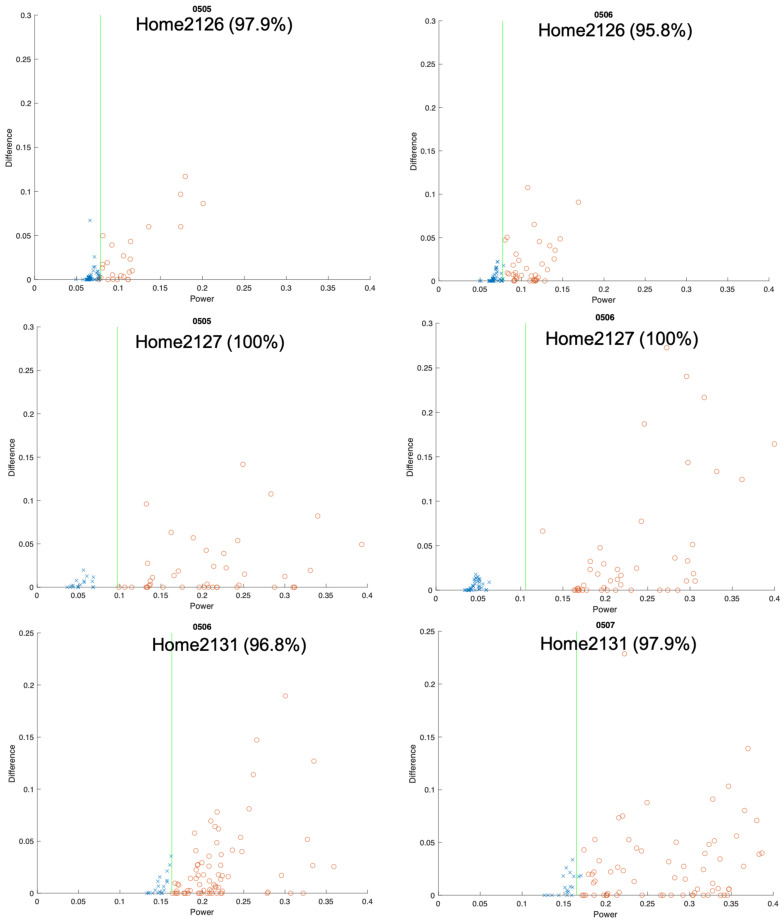
Example threshold accuracy assessment. The green line represents the background power threshold, “x” indicates no appliance usage, and “o” indicates appliance usage.

To validate the accuracy of identifying appliance usage with this background power threshold, this study randomly selected 10 households from the third-party smart meter users, collecting 24 days of power consumption data for each household. This sample size was chosen due to the preliminary testing phase of the methodology, which aims to ensure feasibility before broader implementation. The average of the data from the previous 14 days was taken as the threshold, and a new threshold was generated every day for 10 days. Figure 6 shows the results for accuracy verification, depicting the variation in appliance usage patterns across households, while exhibiting similar trends. Employing the defined background power threshold, after scrutinizing 10 households over 10 days (totaling 100 records) for data interpretation accuracy verification, this study achieved an average accuracy rate of 94.8%. While this result is promising, it is preliminary; further research with a larger and more diverse sample size is necessary to confirm these findings.

### 3.2. Daily Household Appliance Usage Analysis

#### Calculation of Active Score and Norm

**Figure 7:**
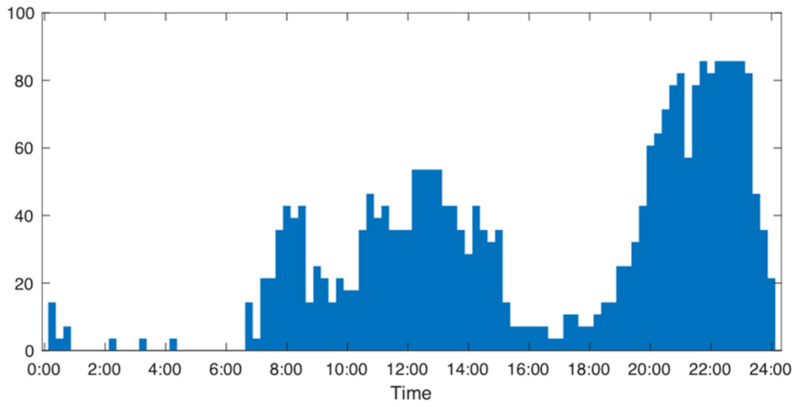
The 28 day norm data for a household with a low norm of 18.0, a norm of 26.3, and a high norm of 34.7.

We calculate the active score and compare it with the user’s historical data (28-day norm) to facilitate daily household appliance usage analysis. The process begins by collecting electricity consumption data from the household using smart meters, calculating the appliance usage threshold, comparing the electricity consumption data with the threshold to obtain periods of appliance usage, and then calculating the active score by determining the percentage of “usage”. This approach offers a comprehensive understanding of the daily activity level, providing invaluable insights into household appliance usage dynamics. For instance, in the data from two depicted households in Figure 3 (Home A and Home B), Home A has an active score of 57.3, whereas Home B records a score of 43.8. The usage data for the current day is compared with the norm data for the previous 28 days to detect any deviations in lifestyle from the established long-term norm. The norm active score is derived by taking the average of the last 28 days’ active scores, and the norm appliance usage by taking the average appliance usage per 15-min period in the last 28 days’ data, expressed as a percentage. Figure 7 illustrates the 28-day appliance usage norm for a household, indicating minimal appliance usage before 7:00 AM and distinct peaks during the morning and noon hours, likely attributed to routine cooking activities. The period from 8:00 PM to 11:00 PM emerges as the most active time, suggesting regular family leisure activities. The “norm active score” represents the average of daily active scores for the last 28 days, while the “high norm” and “low norm” are defined by averaging the activity scores for the most active and least active 14 days, respectively. For example, the norm active score for the depicted household stands at 26.3, with a high norm of 34.7 and a low norm of 18.0.

### 3.3. Assessment of Activity Level and Regularity

This section delves into the utilization of the active score, norm, and correlation coefficients to evaluate the activity and regularity of the user. The active score calculated from the usage for the day is translated into the daily assessment of the household by comparing it to the norm of the last 28 days to obtain the activity level. The low and high norms are calculated from the 28-day norm by sorting the data in ascending order and averaging each of the two halves. This is used to ascertain variations in the household’s daily appliance usage patterns. The appliance usage data (0/1) for the day are compared with the appliance usage norm for the last 28 days to assess the level of regularity of the household.

**Figure 8:**
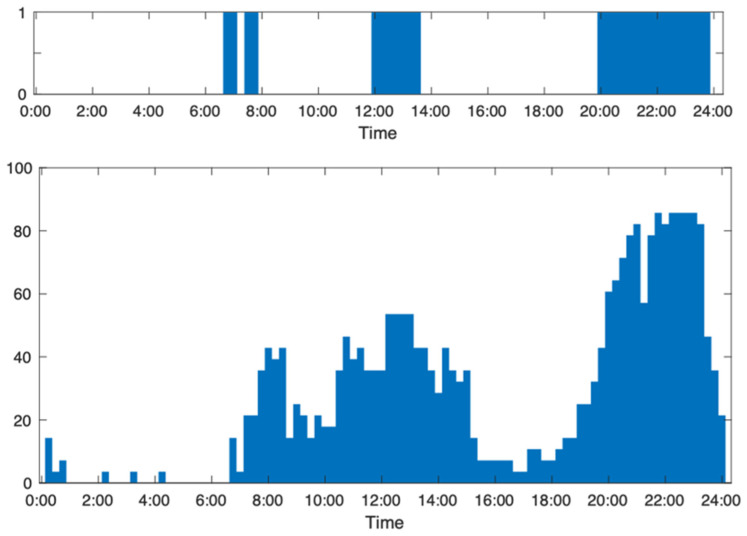
The appliance usage pattern for a day, featuring an active score of 28.1, alongside the previous 28-day norm data for a household, a low norm of 18.0, with a norm of 26.3, and a high norm of 34.7.

The appliance usage (Figure 8, upper part) is generated by converting electricity consumption data into usage (1) and non-usage data (0), as illustrated in Figure 3. As calculated from Figure 8, the active score for the day is 28.1. The active score for the norm is 26.3, with a lower norm of 18.0 and a higher norm of 34.7. The indices of activity based on comparing the active score with the norms are as follows: an active score higher than the high norm indicates a high activity level (level 1); an active score between a high norm and norm indicates a normal activity level; a low activity level lies between the norm and low norm; and abnormally low activity signals an active score that is lower than the low norm. The active score for the household data in Figure 8 is between higher norm and norm, indicating a normal activity level. Regularity is assessed using the correlation coefficients in the household data shown in Figure 8 and categorized them into high regularity for CCs greater than 0.7, normal regularity for CCs between 0.7 and 0.5, low regularity for CCs between 0.5 and 0.3, and irregular for CCs less than 0.3. The CC for the data in the figure is 0.74, suggesting a high level of regularity.

### 3.4. Health Status Dashboard Prototype

**Figure 9:**
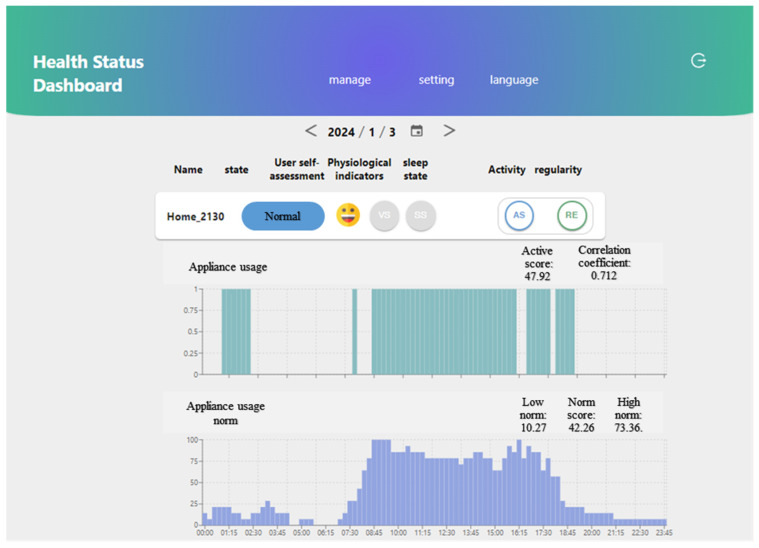
The interface of the health status dashboard for a smart meter household, showing the appliance usage with an active score of 47.92, a correlation coefficient of 0.712, and the appliance usage norm with a low norm of 10.27, a norm of 42.26, and a high norm of 73.36.

In this section, we introduce the implementation of a prototype lifestyle monitoring dashboard, where the indices are put together, analyzed, categorized, and presented to the caregivers and family members to assess the older adults’ health status. The indices for the smart meter usage data include activity and regularity. The users are given a URL to register and gain access to the data. An essential aspect of constructing the health status dashboard is establishing a baseline for each user. The smart meter utilizes data from the last 28 days as the baseline for comparison. The data for the current day are then compared with the 28-day norm data to investigate any deviations from the long-term usage pattern. Figure 9 illustrates the user interface of the health status dashboard. The upper bar chart in the figure displays the appliance usage data for the day (3 January 2024), along with an active score of 47.92 and a correlation coefficient (CC) of 0.712 shown above it. Appliance usage is generated from the electricity consumption data, as described in Section 2. The lower bar chart illustrates the appliance usage norm for the last 28 days (between 4 January 2024 and 31 January 2024), indicating a low norm of 10.27, a norm score of 42.26, and a high norm of 73.36. The health status dashboard categorizes the activity levels into four levels: high, normal, low, and abnormally low. Different colors are utilized to differentiate these levels: green for high, blue for normal, yellow for low, and red for abnormally low activity levels. The user’s activity level is classified as normal, denoted by the blue color on the dashboard. The CC is also employed to assess the regularity of daily electricity usage compared to the 28-day norm, categorized into four levels: high, normal, low, and poor. These categories aid in interpreting the consistency of appliance usage patterns over time. By comparing daily appliance usage with the established norms using the CC, we can identify whether the daily routines closely align with the expected lifestyle or exhibit significant deviations. The CC between the appliance usage data and the appliance usage norm for 28 days shown in the dashboard is 0.712, categorizing the regularity as high. As a result, the status is deemed “Normal”, as indicated by the blue color in the dashboard’s status indicator.

## 4. Discussion

This study investigates how smart meter usage data can be leveraged to monitor the lifestyles of older adults living independently. This study converts energy consumption data into a low-resolution usage/non-usage format to ensure residents’ privacy, while still providing valuable insights into their daily routines and activities. Furthermore, this study explores methods to send these data to caregivers and family members by developing a prototype dashboard, enabling them to monitor the well-being and activity levels of older adults remotely. The dashboard displays the activity and regularity of the user, which are used as measures of the older adults’ well-being. This approach facilitates intervention and support when necessary, contributing to enhanced care and support for independent living [28,29]. Caregivers can benefit from the information derived from smart meter usage data gaining insights into daily activities and routines related to electricity usage. This enables caregivers to detect any deviations or irregularities in behavior that may indicate potential health issues or changes in well-being [30]. Moreover, by having access to objective and quantitative data on the well-being of older adults, caregivers can make more informed decisions and tailor their support strategies accordingly, leading to improved overall care and quality of life [31].

The active scores derived from smart meter data serve as quantitative measures of the household activity level. Higher active scores indicate days with increased energy usage, likely corresponding to the time when occupants are engaged in various tasks or activities. Conversely, low active scores suggest days with reduced energy consumption, potentially indicating more periods of inactivity, which might be a sign of poor health or decreased mobility. By analyzing these scores over time, caregivers and healthcare providers can gain insights into the daily routines of elderly individuals [32,33] and identify any deviations from their usual patterns. Regularity assessments based on smart meter data help identify the consistency and predictability of daily routines. Consistent energy usage patterns indicate stable lifestyles with predictable activity cycles, which can be reassuring indicators of overall well-being. On the other hand, irregularities or fluctuations in energy usage may signal changes in health status, behavioral patterns, or environmental factors. By detecting deviations from the established routines, caregivers can proactively intervene to address emerging issues or provide necessary support [34,35,36]. The interpretation of activity levels and regularity assessments enables the detection of subtle changes that may signify alterations in health or behavior among elderly individuals. For instance, a sudden decrease in activity levels or disruptions in regular patterns could be early indicators of declining health, mobility issues, or cognitive impairment [37]. By continuously monitoring these metrics, caregivers can promptly identify potential long-term health concerns in older adults, which may otherwise lead to institutionalization. This enables them to initiate appropriate interventions, such as medical evaluations or lifestyle adjustments. Armed with this information, caregivers can take proactive measures to address these concerns. They can initiate timely interventions, such as scheduling medical evaluations, adjusting medication regimens, or implementing lifestyle modifications, to support the overall health and well-being of older adults. Additionally, caregivers can use the data to tailor support services and assistance according to the specific needs and preferences of the individuals they care for, thereby enhancing the quality of care provided. Furthermore, the ability to remotely monitor energy usage and activity levels through smart meter data offers caregivers greater flexibility and peace of mind. They can track the well-being of older adults from a distance, ensuring that they remain safe and supported even when the caregivers are not physically present. This remote monitoring capability fosters a sense of security for both caregivers and older adults, promoting independent living while ensuring that assistance is readily available when needed.

**Figure 10:**
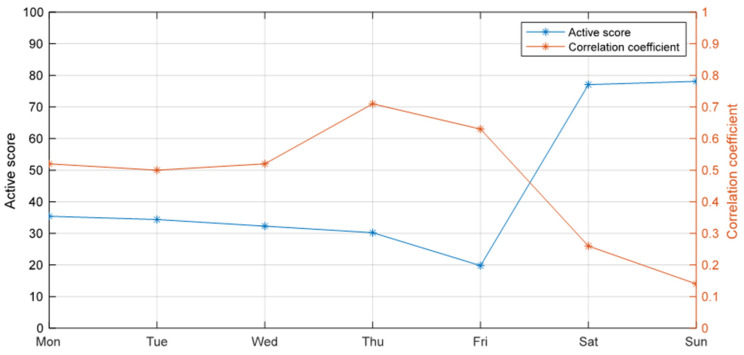
Active score and correlation coefficient data for a user during the week between 12 February 2024 and 18 February 2024.

Household power usage patterns are expected to have small fluctuations during the week, signaling consistent usage behavior by older adults; however, typically, changes are expected during weekends and holidays. Figure 10 illustrates the active score and correlation coefficient (CC) of a user from Monday to Sunday, revealing a notable increase in power usage during the weekend, leading to higher active scores and lower CC values. This trend can be attributed to several factors commonly seen in the lifestyles of older adults. During the week, older adults may adhere to more structured routines, with fewer activities or outings, resulting in lower power usage and higher correlation coefficients as their daily patterns remain consistent. However, they may engage in more leisure activities and social interactions on weekends, including visiting family members, leading to increased power usage and deviations from their regular routines, reflected in higher active scores and lower correlation coefficients. These fluctuations in power usage underscore the importance of comprehensively monitoring older adults’ lifestyles, considering both weekdays and weekends, to capture variations and ensure timely interventions or support when needed. By understanding these patterns, caregivers and healthcare providers can gain insights into older adults’ activities and well-being, allowing tailored interventions and improved support for independent living.

In comparing the accuracy of identifying appliance usage with the background power threshold achieved in this study (94.8%) to those reported in related literature, it is evident that the results are quite competitive. For example, the study on “Effective Load Pattern Classification by Processing the Smart Meter Data” [38] does not specify the exact accuracy rates, but emphasizes the effectiveness of using smart meter data for pattern recognition, which is fundamental in establishing the base load. Similarly, research presented in [39] highlights a methodology that supports high accuracy in load monitoring and appliance identification. This study achieved a notable precision, but did not quantify it in the same direct percentage terms as the current study. Therefore, while direct numerical comparisons are challenging due to the variability in reporting metrics, the approach used in the current study, yielding a 94.8% accuracy rate, stands as highly effective when viewed against the backdrop of methodologies and outcomes discussed in these authoritative sources. This suggests that low-resolution data and the specific methods for threshold calculation in this study are valid and robust for practical applications in lifestyle monitoring based on smart meter data.

While our study has shown promising results, several limitations must be acknowledged. Firstly, our methodology relies solely on electricity consumption data, which may not comprehensively capture all aspects of an individual’s lifestyle. Our approach to establishing a background power threshold and converting data into a low-resolution format may introduce inaccuracies that overlook subtle changes in activity patterns. Additionally, it is important to acknowledge the potential impact of seasonal variations on electricity consumption patterns and their implications for our monitoring system’s effectiveness. The current methodology may not adequately capture how seasonal change influence individuals’ behavior, energy usage habits, and overall lifestyle. Moreover, while the system provides valuable insights into the long-term trends and gradual changes in lifestyle patterns, it may not be suitable for addressing acute health emergencies. Instead, caregivers should view the system as a supplemental tool for proactive health management, enabling them to detect subtle changes in behavior and routine that may signal underlying health concerns. It is essential to emphasize the importance of integrating our monitoring system with the existing health monitoring solutions designed for the real-time detection of acute health events, such as falls or medical emergencies. By complementing each other, these systems can provide a comprehensive approach to caring for older adults living independently, addressing both the long-term trends and immediate health concerns.

The health status dashboard prototype is designed to integrate data from three types of technologies commonly used in home monitoring services: wearable devices, ambient sensors, and household meters. Integrating the data from these sources serves several crucial purposes, but also has limitations. Wearables provide the continuous monitoring of vital signs, offering the advantage of real-time data collection for the immediate detection of abnormalities to provide a timely intervention. However, the drawbacks include potential discomfort or irritation from prolonged wear, the need for regular charging, and limited accuracy compared to that of medical-grade devices. Ambient sensors, such as smart mattress sensors, offer the passive monitoring of sleep patterns, providing valuable insights into sleep quality and duration without requiring active user engagement. They are non-intrusive and can seamlessly integrate into the home environment, but require additional installation. Smart meters track energy consumption patterns and the usage of household appliances, offering indirect insights into daily routines and activities. One advantage is their ability to capture overall energy usage trends and identify deviations from the normal patterns, which may indicate changes in lifestyle or health status. Smart meters are also cost-effective and readily available in many homes, but may lack granularity in capturing specific activities or behaviors. By integrating the data from wearables, ambient sensors, and smart meters, the health status dashboard prototype offers a comprehensive and multi-dimensional approach to monitoring the well-being of older adults, enabling the early detection of health issues, personalized care interventions, and proactive support for independent living.

Sleep data are sourced from the user’s home using the WhizPad system [40], while vital sign data are obtained from dedicated monitoring devices transmitted to a gateway through BLE and stored in a database, with access to the data facilitated through APIs. Data collected from smart meters yield two indices: activity and regularity. Sleep data encompass five indices, the total time in bed, sleep continuity, sleep efficiency, prolonged pressure time, and the total sleep time, which are compared to those of the two-week norm [40]. A vital sign system also provides indices for the heart rate, respiratory rate, blood pressure, and blood oxygen levels.

The health status dashboard includes a crucial section displaying the overall usage status. Specifically, it incorporates a smart meter component that utilizes two key indices. These indices are derived from comparing the previous day’s active score with the last 28-day norm and the correlation coefficient of the preceding day’s appliance usage data with the appliance usage norm of the previous 28-day data. These indices offer insights into the users’ activity and regularity levels, enhancing the understanding of their lifestyles and daily routines. The activity levels are categorized based on the comparison with norms, including a high activity level (an active score higher than high norm), a normal activity level (an active score between norm and high norm), a low activity level (an active score between norm and low norm), and an abnormally low level (an active score lower than low norm). Similarly, the regularity levels are determined based on the correlation coefficient, categorized as high regularity (CC ≥ 0.7), normal regularity (0.5 ≤ CC < 0.7), low regularity (0.03 ≤ CC < 0.5), and irregular (CC < 0.3). Users’ subjective evaluations conveyed through emojis enrich the dashboard’s assessment capabilities. A score from one to four indicates the user’s feelings, with face emojis representing the score; 1 depicts happiness, and 4 indicates sadness. The dashboard employs a color-coded system for interpretation, with colors representing the level of indices and the final assessment of users’ well-being. Green signifies a positive status, blue indicates normal, yellow denotes that attention is required, and red signals abnormality. Gray is used when the data are unavailable. For instance, in Figure 9, the user’s active score for the day falls between the norm score and the high norm, suggesting a normal activity level depicted by the blue color in Figure 9. Additionally, the CC exceeds 0.70, indicating a high regularity level, confirmed by the green color. Vital sign (VS) and sleep status (SS) data are currently unavailable at this phase, as indicated by the gray color. The overall user status shown in the dashboard is normal, aligning with the indices at the highest level (activity).

## 5. Conclusions

In this study, we utilized electricity consumption data collected from smart meter sensors installed in the homes of independently living older adults to track their lifestyle patterns. Processing these data at 15-min intervals provides valuable insights into their daily routines and energy usage behaviors. To safeguard privacy, we employed processing techniques to establish a base load threshold and convert the data into a low-resolution format (0/1), allowing us to focus on activity and regularity assessments. Our methodology involved calculating the active scores, norms, and correlation coefficients to evaluate activity and regularity, providing a comprehensive understanding of older adults’ daily habits and routines. This approach enables us to identify deviations or anomalies that may indicate changes in health or well-being. To facilitate visualization and interpretation, we developed a dashboard prototype that presents the results intuitively and in a user-friendly way.

Moreover, the dashboard is designed to integrate sleep information and vital signs data into our monitoring system, aiming for a holistic approach to elderly care. By incorporating these additional sources of information, we aimed to provide caregivers and family members with a comprehensive overview of older adults’ health status and well-being. While our study has shown promising results in monitoring the lifestyle patterns of older adults using smart meter data, several limitations must be acknowledged. Our methodology relied solely on electricity consumption data, which may not capture all the aspects of an individual’s lifestyle comprehensively. Additionally, it is important to acknowledge the potential impact of seasonal variations in electricity consumption patterns and their implications for our monitoring system’s effectiveness. Changes in seasons can influence individuals’ behavior, energy usage habits, and overall lifestyle, which may not be adequately captured by our current methodology. While the system provides valuable insights into the long-term trends and gradual changes in lifestyle patterns, it may not be suitable for addressing acute health emergencies. Instead, caregivers should view the system as a supplemental tool for proactive health management, enabling them to detect subtle changes in behavior and routine that may signal underlying health concerns.

Looking ahead, our primary objective is to further enhance the monitoring system by integrating vital signs and sleep data, improving the base load detection algorithms, and exploring advanced data analysis techniques. Additionally, real-world system validation and deployment will be crucial steps to ensure the effectiveness and reliability of our monitoring solution in supporting caregivers and keeping family members informed about the health status of older adults living independently.

## Figures and Tables

**Table 1:** Household appliance power ratings.

Appliance	Power (W)	Hr/day	kWh/day
Air condition	2200	8	17.6
Hair dryer	800	0.25	0.2
Rice cooker	1250	0.4	0.5
Dehumidifier	630	4	2.52
Electric fan	70	8	0.56
Microwave oven	1700	0.17	0.289
Hot water kettle	700	0.5	0.35
Refrigerator	200	24	4.8
Electric pot	600	0.67	0.402
Washing machine	500	0.67	0.335
Television	300	8	2.4

## Data Availability

The smart meter data is unavailable due to privacy and Taiwan Power Company ethical restrictions.
